# Serial crystallography using automated drop dispensing

**DOI:** 10.1107/S1600577521006160

**Published:** 2021-07-22

**Authors:** Zhen Su, Joshua Cantlon, Lacey Douthit, Max Wiedorn, Sébastien Boutet, Jan Kern, Chun Hong Yoon, Daniel DePonte

**Affiliations:** a Linac Coherent Light Source, SLAC National Accelerator Laboratory, Menlo Park, CA 94025, USA; b Department of Applied Physics, Stanford University, 348 Via Pueblo Mall, Stanford, CA 94305, USA; c SCIENION AG, Volmerstraße 7a, 12489 Berlin, Germany; d Molecular Biophysics and Integrated Bioimaging Division, Lawrence Berkeley National Laboratory, Berkeley, CA 94720, USA; e Center for Ultrafast Imaging, Universität Hamburg, Luruper Chaussee 149, 22761 Hamburg, Germany; f Center for Free-Electron Laser Science, Deutsches Elektronen-Synchrotron DESY, Notkestraße 85, 22607 Hamburg, Germany

**Keywords:** drop on demand, pulsed sample delivery, serial femtosecond crystallography

## Abstract

Automated, pulsed liquid-phase sample delivery has the potential to greatly improve the efficiency of both sample and photon use at pulsed X-ray facilities. In this work, an automated drop on demand (DOD) system that accelerates sample exchange for serial femtosecond crystallography (SFX) is demonstrated. Four different protein crystal slurries were tested, and this technique is further improved here with an automatic sample-cycling system whose effectiveness was verified by the indexing results. Here, high-throughput SFX screening is shown to be possible at free-electron laser facilities with very low risk of cross contamination and minimal downtime. The development of this technique will significantly reduce sample consumption and enable structure determination of proteins that are difficult to crystallize in large quantities. This work also lays the foundation for automating sample delivery.

## Abstract 2

The development of automated sample delivery and drop on demand methods is described.

## 1. Introduction   

Liquid sample delivery at free-electron laser (FEL) facilities has, to date, been mostly through continuous flow devices and manually exchanged samples and injectors. Several reviews have been written on FEL sample delivery, see for example the work by Bergmann *et al.* (2017 ▸) and Boutet *et al.* (2018 ▸). Droplet dispensing technology has been employed for some FEL applications such as serial femtosecond crystallography (SFX) and hard X-ray spectroscopy in which drop on demand (DOD) dispensers were used (Echelmeier *et al.*, 2020 ▸; Fuller *et al.*, 2017 ▸; Mafuné *et al.*, 2016 ▸; Miller *et al.*, 2019 ▸; Roessler *et al.*, 2016 ▸). Another technology in which drops are generated through controlled breakup of liquid jets has been used for liquid-phase and high-energy density studies (Kim *et al.*, 2018 ▸; Sellberg *et al.*, 2014 ▸). Both methods have seen decades of use in the printing industry with the latter referred to as continuous inkjet (CIJ) printing.

A more recent application of drop dispensing technology to biological fields, especially as a tool for high-throughput screening, has necessitated development of a considerable amount of DOD automation which could be readily adopted to sample delivery efforts at FELs (Echelmeier *et al.*, 2020 ▸; Fuller *et al.*, 2017 ▸). Drop generation for the life sciences is generally achieved by means of acoustic pulses rather than thermal excitation as is common in consumer inkjet printers. Thermal excitation uses a resistive heater to vaporize a small element of fluid creating a pressure pulse needed to eject a drop. An acoustic pulse can be generated by either a piezo device surrounding a converging glass tube or by a transducer behind a liquid surface or converging aperture.

The advantages of DOD technology are greatest for moderate to low repetition rate FELs (<10 kHz), where there can be significant savings in sample consumption using as little as tens of picolitres per drop rather than a continuous flow of tens of millilitres per minute. The CIJ method where jet breakup is driven through Plateau–Rayleigh instability may be a better alternative for high repetition rate (>100 kHz) which is beyond the range of DOD but closer to the natural breakup frequency of most continuous jets used at FELs (Kim *et al.*, 2018 ▸). In either case, the automation of routine tasks such as nozzle cleaning and sample exchanges would be beneficial. Rapid exchange of samples might even allow a single beamline to rapidly screen hundreds of samples per day. In the following, we demonstrate a pulsed sample delivery source that automates many of the tasks currently performed manually.

## 2. Methods   

### 2.1. Drop dispenser   

**Figure 1:**
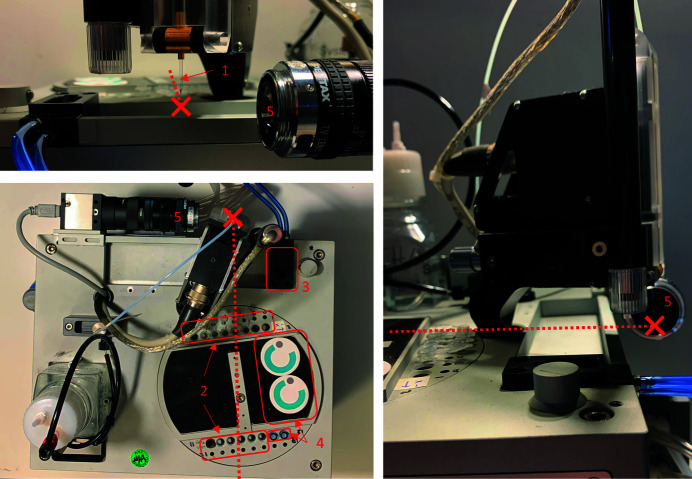
Setup of the dispense system. (1) Nozzle just above the interaction point (marked with red cross), (2) sample and buffer tube holders, (3) tip-wiping station, (4) nozzle-washing station and (5) drop camera. The beam path and interaction point are represented by the red dashed line and red cross, respectively.

A Scienion (sciFLEXARRAYER) drop dispensing system was provided on loan by Scienion AG. The drop dispense system (Fig. 1 ▸) consists of a robotic arm and stage, piezo dispensing capillary (PDC), wash station, inspection microscope, and sample wells that hold micro-eppendorf tubes. The PDC is moved in pre-programed routines between the fill positions at the wells, and the wash station where it is cleaned and inspected. An additional dispensing position was added just above the FEL interaction point. The sample is aspirated through the front of the PDC (drop exit aperture) in volumes ranging from 3 µl to 60 µl, then dispensed into the interaction point in drops of about 250 pl each. After dispensing, the nozzle could be refilled with more of the same sample or washed while sonicating in a water bath before picking up the next sample. The drop dispense system uses a pump to aspirate samples, buffers and cleaning solutions into the nozzle and also to wash samples out of the nozzle. Washing consisted of flushing the nozzle with water while being driven at high frequency in a water bath to wash the nozzle both inside and out. The water bath was also continuously flushed to prevent cross-contamination. The exterior of the nozzle could be cleaned separately by dipping it in the water bath or by wiping the exit surface on a cloth pad. The nozzles are coated to provide a stable hydro­phobic external surface to prevent droplet deviation during prolonged runs. When picking up samples, a sample buffer would be aspirated first to prevent contact of the crystals with the system fluid. The crystal slurry would then be aspirated and dispensed back into the well to resuspend the crystals before being picked up again and taken to the interaction point. All the above operations, cleaning, filling, wiping, dispensing *etc.*, were carried out remotely from the beamline control room. Dispensed drops were spherical and roughly 80 µm in diameter moving at 1.5 m s^−1^. During ejection, a thin strand ‘tail’ of fluid can extend between the drop and nozzle which may extend for several hundred micrometres depending on the properties of the fluid and the pulse shape used to drive the piezo.

### 2.2. Experimental setup   

Droplet-based SFX tests (experiment name: mfx13016) were carried out at the Macromolecular Femtosecond Crystallography (MFX) beamline (Sierra *et al.*, 2019 ▸) at the Linac Coherent Light Source (LCLS), SLAC National Accelerator Laboratory in the standard crystallography configuration using the Rayonix detector at 10 Hz and 9.28 keV photon energy. The dispensing system was too large for the MFX helium enclosure so lead and copper shielding was added to the dispenser to reduce air scatter. The dispenser was modified to place the microscope/camera where it could view the X-ray interaction point. The dispenser model used did not have external triggering capability to accept the FEL timing signal so a driver supplied by Microfab Technologies Inc. was employed to drive both the dispenser and a strobe light used to time the drops with the arrival of X-ray pulses. All samples loaded into the dispenser reservoir wells were kept at ambient temperature for the duration of the shift.

### 2.3. Samples   

Four different crystal samples were tested. Proteinase K and thaumatin crystals were bipyramidal and 10–30 µm with proteinase K also having a second size distribution of microcrystals in the 2–3 µm range in the same crystal slurry. Xylanase crystals were 10–30 µm rhombohedral plates with a smaller number of needles 10–50 µm long and 2–5 µm wide. Alcohol de­hydrogenase also formed needle-shaped crystals 7–15 µm long and 2–3 µm wide. The number densities for thaumatin and xylanase were similarly estimated at 400 crystals nl^−1^ with proteinase K and alcohol de­hydrogenase estimated at 40–50 crystals nl^−1^. All samples excluding alcohol de­hydrogenase were previously tested and showed diffraction to better than 2 Å at Advanced Light Source BL8.2.1.

Protein crystals would settle to the bottom of the sample wells over time but could be resuspended by the device just prior to uptake. First, 25 µl of buffer was aspirated into the nozzle. Then 3 µl of sample was taken up and quickly dispensed back into the sample well without piezo actuation. This was repeated three times to mix the sample before the final volume, typically 5 µl, was taken up for dispensing into the X-ray probe.

As an additional test of crystal damage, a small volume of thaumatin was cycled through the dispenser prior to beam time. A few millilitres of sample were aspirated into the nozzle then dispensed into a micro-eppendorf tube at 1.2 kHz.

### 2.4. Hit finding and indexing   

Hit finding based on Bragg reflections and detector geometry correction were performed using *Psocake* (Thayer *et al.*, 2017 ▸; Yoon, 2020 ▸). Peak finding parameters for all datasets classifying a hit were as follows: a minimum pixel count of 2 above adu-threshold of 100 with a minimum signal-to-noise ratio of 7 was considered a peak, and an image containing at least 15 peaks was classified as a crystal hit. The diffraction patterns display strong diffraction rings from lead and copper shielding used to reduce air scatter. To prevent the lead and copper diffraction rings from contributing to the hit rate, we manually masked out these rings in each run before the peak-finding step.

The crystal hits were then indexed using *indexamajig* in *CrystFEL* (White *et al.*, 2012 ▸, 2016 ▸) using the peaks found with *Psocake*. Hits found were indexed using *MOSFLM* (Powell *et al.*, 2013 ▸), *DirAx* (Duisenberg, 1992 ▸), *XDS* (Kabsch, 2010 ▸) and *XGANDALF* (Gevorkov *et al.*, 2019 ▸) algorithms with indexing tolerances of 5% for lattice lengths and 1.5° for angles, and integration radii were set to 3, 4, 5 and the ‘--multi’ option was switched on to enable indexing of multiple crystal lattices in a single image. The diffraction distance was optimized so that the histograms of indexed crystal lattice constants were close to Gaussian distributions. The detector center was optimized using *detector-shift* in *CrystFEL*. The hit-finding and indexing statistics are summarized in Table 1 ▸.

## 3. Results   

### 3.1. Hit and indexing rate   

The automation ran efficiently. The tests were carried out in a single 12 h shift most of which was used to bring the X-rays to the interaction point and install shielding. Once the sample delivery system was set up and running things progressed fairly smoothly; in the remaining 137 min, there were 13 sample exchanges or reloads. Each reload aspirated enough sample for up to 10 min of run time. A full wash and reload as described in *Methods* [Sec sec2] took about 3 min from data collection. If the sample was reloaded with identical samples and no cleaning was needed, a little under 1 min was required.

**Figure 2:**
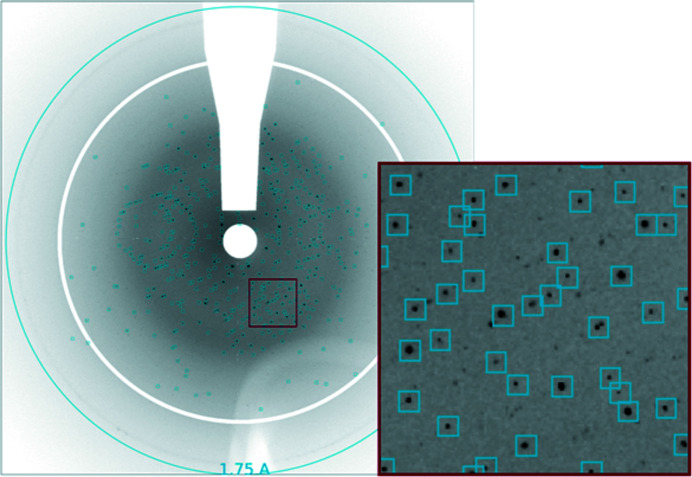
Diffraction pattern from a single drop containing multiple thaumatin crystals (run 31). Blank areas represent masked-out pixels where shadow from the nozzle and diffraction from copper shielding are removed prior to peak finding. Dark spots represent Bragg peaks, and blue squares indicate peaks found by *Psocake*.

**Figure 3:**
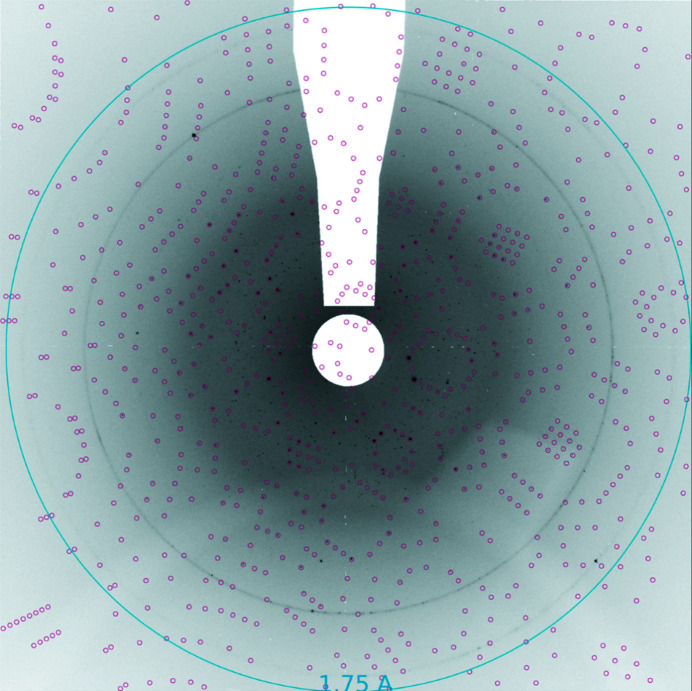
Diffraction pattern from thaumatin and predicted spots from indexing (run 31). Black dots are possible Bragg peaks, and red circles indicate predicted Bragg peak positions indexed by *indexamajig* in *CrystFEL* (White *et al.*, 2012 ▸, 2016 ▸).

Three of the four samples (except alcohol de­hydrogenase) produced nice diffraction patterns as shown in Figs. 2 ▸–4 ▸ ▸ for thaumatin. It is not known why alcohol de­hydrogenase did not diffract. It was run for a few minutes at the end of the shift and there was not time to try an additional preparation. It may have been damaged by the dispenser, but it was also the only sample that had not been tested at a synchrotron prior to the FEL beam time and so may not have been of diffraction quality. For the higher density slurries, patterns generated from multiple crystals were common as shown in the diffraction pattern in Fig. 2 ▸ identifying crystal peaks. Figs. 3 ▸ and 4 ▸ show thaumatin diffraction patterns with indexed Bragg peaks identified. Red circles show the predicted positions of the Bragg peaks where we integrated Bragg intensities. The strong diffraction rings were caused by scattering from the copper shielding at 2.09 Å and 1.81 Å.

**Figure 5:**
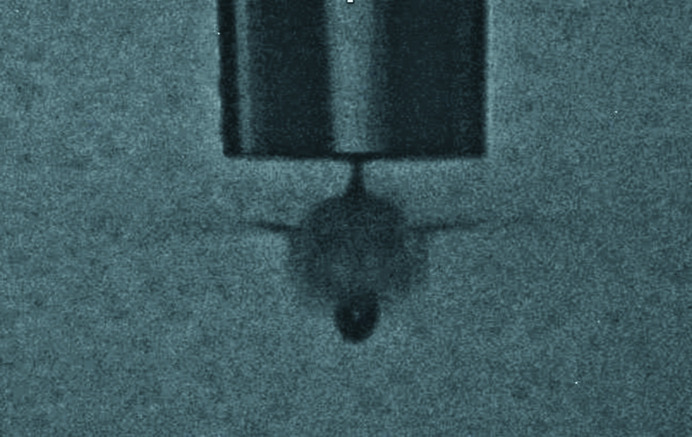
Tail of the droplet shown exploding as it is intercepted by an FEL pulse.

**Figure 6:**
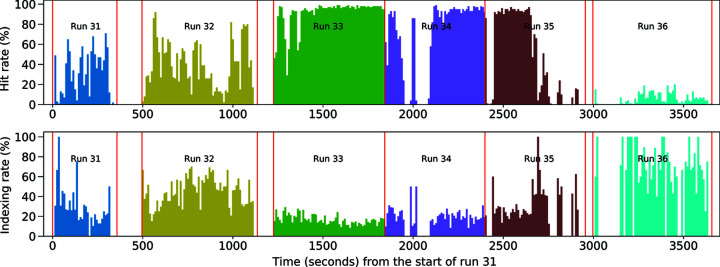
Continuous hit and indexing rates from the start of run 31 to the end of run 36. The data are colored based on run numbers and divided by red vertical lines indicating the start and end time points of each run. Neighboring runs being divided by two red vertical lines indicate an interval between these two runs; for example, there is a 139 s time interval for wash between runs 31 and 32. Each small time bin is 10 s long. The hit rate is defined as the percentage of hits identified by *Psocake* among all diffraction patterns collected in each time bin, and the indexing rate here is the ratio of indexed events to the number of hits in each time bin, with multiple-crystal hits counted once.

Hit and indexing rates are shown in Table 1 ▸ and varied with drop stability but were otherwise consistent with expectations for concentration. Drop stability was affected by the build-up of sample debris on the nozzle face. This required constant effort from the operator to keep the droplet stream aligned within the X-ray focus as well as occasional nozzle cleaning. The hit rate could be temporarily improved by shooting the long thin tail behind the drop as shown in Fig. 5 ▸; however, this increased the frequency with which the nozzle required cleaning. Runs 31–36 in the table show varying hit rates ranging from 90.9% in run 33 to 5.5% in run 36. The hit rate, defined as the fraction of patterns where 15 peaks or more were found, and the indexing rate, defined as the ratio of indexed events to the number of hits, are shown for a continuous stretch of ∼3600 s starting from run 31 in Fig. 6 ▸. When the dispenser was aligned and timed to the FEL pulses, the hit rate was fairly constant at about 90% with occasional dips due to stability and longer pauses for nozzle washing and reloading. The roughly constant maximum hit rate through runs 33–35 arising from constant sample concentration infers that there is no settling issue. Towards the end of run 35, crystals began to run out and by run 36 were fairly rare. Sample buffer is loaded before sample and the decrease in hit rate towards the end of the sample is expected. The indexing rate for run 36 was higher than 33 through 35, which is consistent with fewer multiple-crystal hits.

In order to check whether the automated drop dispenser setup could be prone to cross contamination of the sample, we indexed all the runs with unit cells from all samples present at the experiment (as summarized in Table 1 ▸). We noticed that a few patterns were indexed as alcohol de­hydrogenase for runs not containing alcohol de­hydrogenase. Upon careful inspection, these diffraction patterns were indexed correctly using the unit cell of the corresponding run. The integrated Bragg spots indexed using the alcohol de­hydrogenase unit cell, however, did not contain actual Bragg spots indicating that alcohol de­hydrogenase was not the diffracting crystal. Other indexed patterns with incorrect unit cells were also confirmed to be misindexed, with most predicted spots not matching actual Bragg peaks, hence ruling out any cross contamination.

### 3.2. Sample damage test at 1.2 kHz   

**Figure 7:**
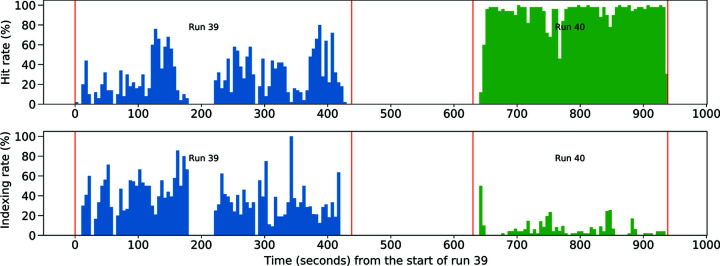
Continuous hit and indexing rates from the start of run 39 to the end of run 40. The data are colored based on run numbers, with the start and end time points of each run labeled with red vertical lines. Each small time bin is 5 s long, and there is a 192 s time interval between these two runs. The hit and indexing rates are calculated using the same method described in Fig. 6 ▸.

**Figure 8:**
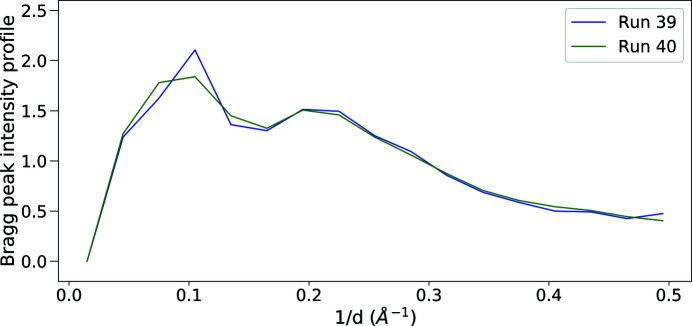
Radially averaged Bragg peak intensity profiles up to ∼2 Å for run 39 and 40. The per-image radial profile of a single pattern was calculated by averaging Bragg peaks identified by *Psocake* in each shell, which was further scaled to minimize the *L*_2_ distance to the reference radial profile of the first hit in the same run. The radial profile curve of a whole run, as shown in this plot, was calculated by averaging these scaled per-image radial profiles of all hits in this run. For improved comparison, both curves of run 39 and 40 are scaled to have a mean value of 1.

As a test of sample damage due to dispensing, sample was dispensed twice and compared with the single-dispensed sample. A small quantity of thaumatin was aspirated and dispensed at 1.2 kHz into a microwell prior to beam time. Table 1 ▸ shows a 25.3% hit rate and 39.1% indexing rate for the cycled sample in run 39, and a much higher hit rate (88.5%) and lower indexing rate (5.3%) for the fresh sample immediately after in run 40. The continuous hit and indexing rates are shown in Fig. 7 ▸. The hit rate was lower for the cycled sample likely due to a lower concentration as implied by the higher indexing rate. The concentration of the cycled sample was not recorded and so the differences in hit rate may be due to initial concentration. As a better measure of sample quality, the radially averaged Bragg peak intensity profiles of run 39 and 40 are compared in Fig. 8 ▸. To calculate the radial profile of a certain run, Bragg peaks identified by *Psocake* in each hit pattern were averaged in the uniformly divided resolution shells, giving rise to the per-image radial profile which was further scaled to minimize the *L*_2_ distance to the reference profile of the first hit in this run. The radially averaged Bragg intensity profile of a whole run was then calculated by averaging these scaled per-image radial profiles of all hits in this run. For improved comparison, both radial profiles of run 39 and 40 in Fig. 8 ▸ were scaled to have a mean value of 1. Comparison of these two radial profile curves shows little difference up to ∼2 Å, implying that the sample quality is not diminished by operation at 1.2 kHz. Moreover, Table 1 ▸ presents three additional statistics: the average number of Bragg peaks among all hits in each run, the average intensity of all Bragg peaks identified by *Psocake* in each run and the average diffraction resolution limit extracted from the *CrystFEL* (White *et al.*, 2012 ▸, 2016 ▸) indexing results of all indexed crystals in each run. All this information shows that run 39 and 40 are similar in all aspects with the exception that run 40 has more diffracted Bragg peaks which might arise from the higher sample concentration and lead to more multiple-crystal hits that are hard to be indexed.

## 4. Discussion and conclusions   

Automated, pulsed sample delivery shows potential utility for sample delivery at FEL facilities. Four different crystal slurries were evaluated, where proteinase K, thaumatin and xylanase were previously tested and showed diffraction at synchrotron light sources, and these three crystal samples also diffracted well at LCLS with no signs of damage for thaumatin that had previously been dispensed at higher frequency.

Liquid volume consumption as measured by volume per drop was 250 pl per drop or 0.9 µl min^−1^ at our repetition rate of 60 Hz. This has the potential to be reduced by using smaller drops; we used spherical 80 µm drops for this study but piezo actuated dispensers can operate in the 40 µm to 50 µm range and, with decreased repeatability, even lower. Using smaller drops and higher frequency, the crossover at which volume flow is comparable for drops and continuous flow from a gas accelerated cylindrical jet is about 10 kHz. For hard X-ray spectroscopies requiring a similar interaction volume from either continuous or pulsed flow, the total required sample volume for continuous flow will always be higher. Volume flow rates alone are an insufficient measure of efficiency for SFX; hit rate and indexing rate must also be considered. Our indexing rate was relatively low but not unexpected due to the large number of multiple hits. A larger volume of sample is exposed per shot when using a droplet source and we did not run dilution series to find the optimal operating concentration. Running drops that are larger in diameter than a typical SFX continuous jet have a twofold effect on sample consumption: (1) a beneficial effect of reduced sample concentration and (2) a detrimental effect of increased scattering from the excess fluid which reduces the hit and indexing rates. How this is balanced is too heavily dependent on sample preparation to make a quantitative comparison. It is clear however that there is far more opportunity to optimize operating conditions, such as crystal size, concentration, interaction volume, *etc.* when using drops than when using a continuous jet for which small crystal size and maximum concentration are needed.

Sample settling did not appear to be a problem. Sample exchanges and nozzle cleaning were conducted remotely and efficiently within a few minutes. The drop dispensing system was reconfigured to access and image droplets at the interaction zone outside the robotic enclosure. With more advanced systems, samples can be exchanged faster and far more samples can be held in the system in multiple microwell plates. In addition to sample exchanges, nozzles can be remotely exchanged as well. Together this should make high-throughput SFX screening possible at FEL facilities with very low risk of cross contamination and minimal down time.

While drop dispensing may have some advantages for sample consumption at low (<10 kHz) repetition rate, the larger advantage may lie in its potential to be automated. Sample exchanges and nozzle cleaning have heretofore been carried out manually. Although this drop dispense system was an older model with limited functions, its front-loading, aspirating nozzles showed the potential to automate these tasks and others. Use of microwell plates in more recent, higher capacity, higher speed, temperature- and humidity-controlled systems will allow for perhaps a few hundred samples to be tested at a single 12 h 120 Hz shift without human intervention. Crystallization screens to determine optimal crystallization conditions could then be carried out at the beamline with results given by a combination of visualization and X-ray scattering. The system tested here as well as modern microwell systems are limited to operation in a humidity-controlled system and so neither screening nor nozzle washing are expected to be possible under vacuum. The second point is also relevant to single-sample SFX experiments – as with continuous jets, debris accumulation on the nozzle exit due to sample explosion caused some drift in the drop position, reducing hit rates. The automated wash routine is very useful in that regard. Overall, automation also has the potential to make SFX less labor-intensive with a single investigator remotely operating the dispense system to perform tasks otherwise carried out by beamline staff.

## Figures and Tables

**Figure 4:**
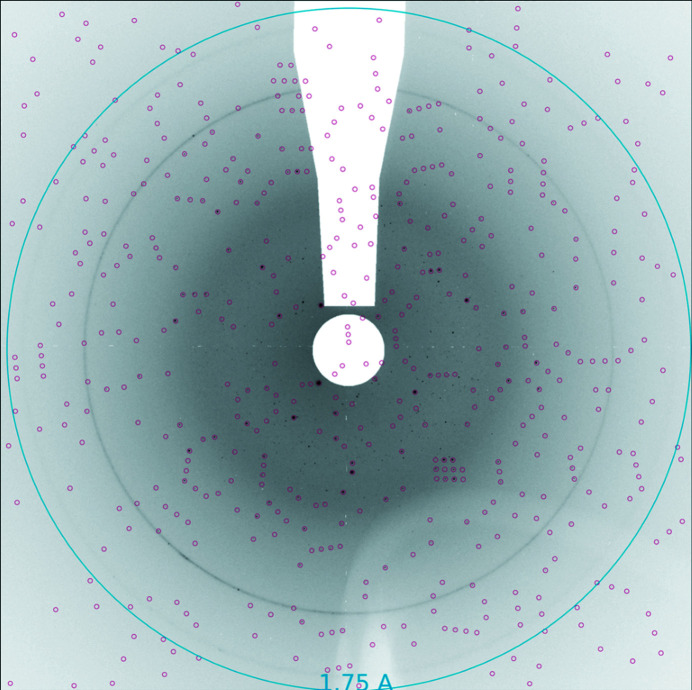
Diffraction pattern from thaumatin and predicted spots from indexing (run 39). Black dots are possible Bragg peaks, and red circles indicate predicted Bragg peak positions.

**Table 1:** Experiment details and data analysis results of each run

Run number	31	32	33	34	35	36	37	38	39	40	42	43	44	45
Sample	Th†	Th	Th	Th	Th	Th	PK†	PK	Th	Th	Xy†	AD†	AD	AD
Run time (s)	357	640	617	556	557	660	733	729	438	309	129	58	84	74
Number of hits	1083	2454	5604	3756	2480	363	683	771	1109	2732	670	0	0	1
Hit rate (%)	30.4	38.3	90.9	67.6	44.5	5.5	9.3	10.6	25.3	88.5	51.9	0	0	0.1
Number of indexed‡ (Th)	271	1094	963	770	726	266	0	0	434	145	1	0	0	0
Number of indexed (PK)	0	0	0	0	1	0	569	709	0	0	0	0	0	0
Number of indexed (Xy)	0	0	0	0	0	0	0	0	0	0	41	0	0	0
Number of indexed (AD)	1	8	4	3	4	0	5	11	3	1	2	0	0	0
Indexing rate§ (%)	25.0	44.6	17.2	20.5	29.3	73.3	83.3	92.0	39.1	5.3	6.1	0	0	0
Average diffraction limit (Å)	3.1	3.2	3.0	3.1	3.2	3.4	2.7	2.7	3.1	2.9	2.6	–	–	–
Average number of Bragg peaks	82	56	91	84	64	27	52	67	98	256	90	–	–	17
Average Bragg peak intensity (adu)	970	947	920	897	906	926	2149	2483	1117	1107	1848	–	–	1710

† Th = thaumatin; PK = proteinase K; Xy = xylanase; AD = alcohol de­hydrogenase.

‡ The number of indexed patterns counts multiple crystals in one diffraction pattern.

§ The indexing rate here is defined as the ratio of indexed crystals to the number of hits using the correct unit cell.
